# Fetal inflammatory response and risk for psychiatric disorders

**DOI:** 10.1038/s41398-023-02505-3

**Published:** 2023-06-24

**Authors:** Blake Gibson, Eli Goodfriend, Yongqi Zhong, Nadine M. Melhem

**Affiliations:** 1 University of Pittsburgh School of Medicine, Pittsburgh, PA USA; 2 University of Pittsburgh Medical Center, Pittsburgh, PA USA; 3 The Johns Hopkins University Bloomberg School of Public Health, Baltimore, MD USA

**Keywords:** Pathogenesis, Predictive markers

## Abstract

Inflammation contributes to numerous neuropsychiatric disorders, especially those that first appear in childhood. Maternal intrauterine environment, including the placenta, has a role in brain development and risk for neuropsychiatric disorders. This study examines the link between fetal inflammatory syndrome (FIRS), which is placental inflammation in the peri-partem period, and neuropsychiatric disorders during childhood.This is a retrospective cohort study using data from electronic medical records over a 19-year period at one women’s hospital. The study includes 4851 children born with placentas meeting criteria for and 31,927 controls identified with normal placentas born during the same period. To be diagnosed with FIRS placenta must contain chorionic vasculitis and/or funisitis. Children had to be in study period for at least 5 years. The primary outcome of the study is incidence of neuropsychiatric disorders during childhood. The secondary outcomes were psychiatric medications prescribed, and psychiatric hospitalizations and treatment. Children born to placentas meeting criteria for FIRS were more likely to be diagnosed with neuropsychiatric disorders (OR = 1.21, CI 95% [1.09,1.35]). Specifically, they were more likely to be diagnosed with autism spectrum disorder (OR = 1.35, CI 95% [1.08, 1.67]), ADHD (OR = 1.27, CI 95% [1.07, 1.49]), conduct disorder (OR = 1.50, CI 95% [1.24, 1.81]), PTSD (OR = 2.46. CI 95% [1.21, 5.04]), adjusting for maternal history of psychiatric disorders, intra-partem substance use, and prescriptions of anti-inflammatory drugs. Children born with placental inflammation are at an increased risk to develop neuropsychiatric disorders. This has profound implications for future research, and early detection, monitoring, and treatment in these children.

## Background

Recent genome-wide association studies and cross-disorder genetic studies show a shared genetic liability across psychiatric disorders with a childhood onset, specifically, autism spectrum disorders (ASD), attention deficit hyperactivity disorder (ADHD), schizophrenia, bipolar disorder (BPD), and major depressive disorder (MDD) [1]. These studies have also implicated an enrichment of genetic loci that have an important role in immune responses. Inflammation (manifested as elevated pro-inflammatory interleukins (e.g. IL-6), a decrease in anti-inflammatory interleukins (e.g. IL-10), or elevated C-reactive protein, TNF-alpha, etc.) has also been linked to a wide range of neuropsychiatric disorders that first present in pediatric populations including ADHD [2], BPD [3], MDD [4], ASD [5, 6], suicidal behavior [7, 8] and schizophrenia [9]. Environmental factors during pregnancy have also been linked to psychiatric outcomes and increased inflammation in offspring and these include maternal smoking [10], toxins/drugs [10], nutrition [10], psychosocial stress [11], infection [12, 13], and intrauterine growth restriction [14]. As a result, there has been increased attention to intrauterine factors in order to improve early detection and treatment of psychiatric disorders in pediatric populations [15].

The developing brain is influenced in-utero by the placenta [16]. There are reciprocal interactions of the immune system between the mother and fetus through the placenta [17–20]. While the neural development in the first two trimesters is driven largely by genetic factors, by the third trimester prenatal/postnatal brain development becomes increasingly influenced by environmental factors. Maternal or fetal inflammation is one of the mechanisms implicated in the long-term alteration of brain development throughout childhood [21–23]. There are two components to placental inflammation, maternal and fetal. Maternal inflammation is defined as acute or chronic chorioamnionitis. This denotes inflammation of the chorion and amnion, which are considered maternal surfaces of the placenta [24]. Fetal Inflammatory Response Syndrome (FIRS) is a condition characterized by systemic activation of the fetal innate immune system and conceptualized as analogous to systemic inflammatory response syndrome (SIRS) in adults [25]. FIRS consists of either funisitis or chorionic vasculitis. Funisitis is an inflammatory process that involves the umbilical cord including the umbilical vein, artery, and Wharton’s jelly [24]; whereas chorionic vasculitis involves fetal vessels of the chorionic plate or the umbilical cord [24]. FIRS can be diagnosed using cord blood and/or histologic examination of the placenta. A proof-of-concept study looking at the histological correlates of FIRS showed IL-6 concentrations to be associated with fetal inflammatory response. Indeed, funisitis and/or chorionic vasculitis showed higher IL-6 levels than chorioamnionitis or normal placental tissue [26]. Thus, the hallmark of FIRS is increased IL-6 concentrations in the placenta [27, 28]. To meet criteria, plasma Il-6 in fetal cord blood must be greater than 11 ng/ml [29]. Since plamsa IL-6 is not routinely done clinically, the presence of funisitis or chorionic vasculitis histologically is indicative of FIRS [29]. FIRS rarely occurs before the 20th week of gestation [30], occurs in 25–40% of preterm births [31], and 4–6% of term births [32]. There are numerous infectious and non-infectious etiologies of FIRS including fetal viral infections such as cytomegalovirus [29], preterm labor with intact membranes [29], and preterm pre-labor rupture of the membranes [29]. Duration of labor was also found to be a risk factor for FIRS [33].

FIRS has been associated with numerous negative fetal and pediatric outcomes including multiple organ dysfunction, septic shock, and death. Several fetal organs appear to be targeted by FIRS including the hematopoietic system, adrenals, heart, lungs, skin, and the brain [24, 29]. Specific to the brain, FIRS has been associated with cerebral palsy [34], periventricular leukomalacia [35], and white matter lesions [36]. While preterm birth and chorioamnionitis are risks for adverse fetal outcomes, FIRS is recognized as a distinct process [26, 29, 31]. To our knowledge, there are no studies examining the risk of neuropsychiatric disorders associated with FIRS. In this study, we examine the association between FIRS and the risk for neuropsychiatric disorders in children and adolescents. We hypothesized that FIRS will be associated with increased neuropsychiatric disorders in childhood in general and with childhood disorders in specific such as developmental delays, intellectual disability, ADHD, and ASD.

## Methods

Electronic health records (EHR) were queried, using honest broker, at one women’s hospital at the University of Pittsburgh Medical Center, Pittsburgh, Pennsylvania after IRB approval. Because we hypothesized an increased risk of childhood neuropsychiatric disorders, we requested EHR for all individuals born between 1/1/1999 and 4/1/2018 with pathology reports with a diagnosis funisitis and/or chorionic vasculitis and thus considered to meet histologic criteria of FIRS. Placentas are not routinely examined after delivery unless clinical suspicion of abnormalities, or poor newborn health, is identified. There were 4851 children born with placentas meeting this criterion during this period. We randomly selected a subset of children born during this period, selecting 31,927 controls who either did not have placenta reports, or whose reports did not mention funisitis, chorionic vasculitis, or chorioamnionitis and thus reflecting a normal placenta. Abnormalities that are non-inflammatory in nature and unrelated to the inflammation examined in FIRS cohort were included in the control group. The placenta pathology report date was used as a surrogate marker for date of birth of the child. For our analysis, we required a follow-up period in the EHR of at least 5 years in order to capture pediatric onset psychiatric disorders. This resulted in 2055 FIRS cases and 13,022 controls with an average length of follow up of 7.7 years (IQR = 2.72), and 7.5 years (IQR = 2.67), respectively. ICD-9 and ICD-10 psychiatric diagnostic codes and medications prescribed were obtained for each child longitudinally throughout the University of Pittsburgh Medical Center (UPMC) system up until April 2018 when the data was queried. Because parental history of psychiatric disorders and maternal substance use are associated with increased risk of psychiatric disorders in their offspring, we also collected maternal EHR data from the UPMC system to examine maternal psychiatric diagnosis, suicidal behavior, and substance use as potential confounders and linked maternal diagnostic and substance use codes to children’s data. If the maternal substance use diagnostic code was rendered within 320 days of placental report date, it was considered intra-partem substance use. Psychiatric diagnostic classification changed significantly between ICD-9 and ICD-10. For a full list of groupings of diagnostic codes, please see Table A1 in the appendix. Records were considered duplicates and dropped if they had similar data on the same day. For example, if the same subject had two records with the same diagnosis code on the same day, these were considered duplicates. We also examined prescriptions by class and used psychiatric medication or psychiatric treatment (inpatient/outpatient psychiatric treatment) as secondary outcomes and as proxies for psychiatric disorder as some providers are reticent to give a psychiatric diagnosis to children for insurance or stigma reasons. Their distributions can be found in Table A2 in appendix.

### Statistical analyses

We compared children born with FIRS and those born without FIRS placentas on individual psychiatric diagnoses, any psychiatric disorders, and secondary outcomes using Chi-square tests and on number of psychiatric inpatient and outpatient encounters using t-tests. Statistical significance was considered at *p* < 0.05. Logistic regressions were also used to examine the differences between children born with FIRS and those born without FIRS placentas on psychiatric diagnoses, adjusting for potential confounders regardless of their statistical relationship with FIRS or with psychiatric diagnoses (e.g., maternal psychiatric diagnoses). Nelson-Aalen cumulative hazard estimators using Cox proportional hazards regression were conducted to examine the differences in time to onset of psychiatric diagnoses following birth between children born with and without FIRS. To examine the potential effects of missing data and years of data available on our results, we conducted sensitivity analyses using all available data and using those with complete data. E.G. conducted the analysis for this manuscript.

## Results

In the univariate analyses, we found children born with FIRS were more likely to have ADHD, ASD, conduct disorders, PTSD, and any psychiatric diagnoses compared to children without FIRS (Table 1). When looking at secondary outcomes, similar univariate associations were found for any psychiatric medications, ADHD medications generally (stimulants only, noradrenaline reuptake inhibitors and alpha 2 receptor antagonists), and narcotic ADHD medications specifically (Table A2). However, there were no significant differences between children born with and without FIRS in the use of anti-anxiety medications, mood stabilizers, antipsychotics and anti-depressants, as well as in psychiatric encounters.

**Table 1:** Distribution of psychiatric diagnoses in children with and without Fetal Inflammatory Response Syndrome (FIRS).

	FIRS (*N* = 1641)	Controls (*N* = 12492)	χ2	*p*	Odds Ratio	Odds Ratio CI
Attention Deficit Hyperactivity Disorder (ADHD)	11.3% (185)	9.1% (1140)	7.62	0.006	1.27	(1.07, 1.49)
Adjustment disorders	5.6% (92)	5.2% (648)	0.43	0.511	1.09	(0.87, 1.36)
Anxiety	6.6% (109)	5.5% (685)	3.46	0.063	1.23	(1.00, 1.51)
Autism Spectrum Disorder (ASD)	6.3% (103)	4.7% (592)	7.0	0.008	1.35	(1.08, 1.67)
Conduct disorders	8.5% (140)	5.8% (730)	17.67	<0.001	1.50	(1.24, 1.81)
Depressive disorders	0.6% (10)	0.5% (61)	0.22	0.641	1.25	(0.64, 2.44)
Obsessive Compulsive Disorders (OCD)	0.2% (4)	0.4% (46)	0.33	0.564	0.66	(0.24, 1.84)
Posttraumatic Stress Disorder (PTSD)	0.6% (10)	0.2% (31)	5.35	0.021	2.46	(1.21, 5.04)
Developmental delay	15.4% (253)	13.7% (1712)	3.41	0.065	1.15	(0.99, 1.32)
Intellectual disability	0.7% (11)	0.3% (43)	3.24	0.072	1.95	(1.01, 3.80)
Any psychiatric disorders	36.1% (593)	31.9% (3980)	11.92	0.001	1.21	(1.09, 1.35)
Suicide attempt	0.1% (1)	0.0% (1)	0.35	0.554	7.62	(0.48, 121.83)

We next examined maternal history of psychiatric disorders, maternal substance use during pregnancy, maternal history of suicidal behavior, and prescriptions of anti-inflammatory drugs as potential confounders (Table 2). There was no difference in maternal history of psychiatric disorders, maternal history of suicidal behavior, or prescriptions of anti-inflammatory drugs between children born with FIRS compared to those born without placentas with FIRS. However, in utero exposure to any substance, and specifically alcohol, cannabis, and nicotine, was significantly increased for children born with FIRS. Neonatal substance use disorder diagnosis was also significantly increased in those with FIRS compared to those without FIRS.

**Table 2:** Distribution of secondary outcomes and potential confounders in children with and without Fetal Inflammatory Response Syndrome (FIRS).

	FIRS percent (*N*)	Control percent (*N*)	χ2	*p*	Odds ratio	Odds ratio CI
*Medications*	*N* = 1717	*N* =10915				
Anti-inflammatory medications	49.6% (852)	47.3% (5168)	2.98	0.084	1.10	(0.99, 1.21)
Anti-infection medications	83.9% (1440)	84.7% (9243)	0.69	0.405	0.94	(0.82, 1.08)
*Maternal history of psychiatric diseases*	*N* = 1631	*N* = 12414				
Any psychiatric disorder in mother	63.4% (1034)	62.1% (7713)	0.93	0.335	1.06	(0.95, 1.17)
Mother had suicide attempt	1.7% (28)	1.2% (147)	2.9	0.088	1.46	(0.97, 2.19)
*In utero exposure to substances*	*N* = 2042	*N* = 12931				
Alcohol exposure in utero	1.3% (27)	0.8% (101)	5.47	0.019	1.70	(1.11, 2.61)
Amphetamine exposure in utero	1.6% (32)	1.2% (158)	1.41	0.235	1.29	(0.88, 1.89)
Cannabis exposure in utero	6.3% (128)	4.1% (534)	18.6	<0.001	1.55	(1.27, 1.89)
Cocaine exposure in utero	1.4% (29)	1.1% (137)	1.78	0.183	1.35	(0.90, 2.01)
Hallucinogen exposure in utero	0.0% (1)	0.0% (2)	0.02	0.878	3.17	(0.29, 34.95)
Nicotine exposure in utero	12.4% (254)	10.4% (1342)	7.65	0.006	1.23	(1.06, 1.42)
Opioid exposure in utero	4.8% (99)	3.9% (506)	3.74	0.053	1.25	(1.00, 1.56)
Sedative exposure in utero	0.7% (14)	0.8% (101)	0.1	0.747	0.88	(0.50, 1.54)
Any substance exposure in utero	12.1% (248)	9.0% (1168)	19.59	<0.001	1.39	(1.20, 1.61)
*Neonatal substance use diagnosis*	*N* = 1641	*N* = 12492				
Neonatal substance use diagnosis	18.6% (305)	12.2% (1526)	51.6	<0.001	1.64	(1.43, 1.88)
*Demographics*	*N* = 2055	*N* = 13022				
Year of birth	2010.6 + /−1.8	2010.8 + /−1.8	t = −4.49	<0.001		

We next examined the relationship of FIRS with psychiatric outcomes after controlling for potential confounders significantly different between those with and without FIRS using logistic and Cox proportional hazards regression models. We used in utero exposure to any substance rather than specific substances to avoid collinearity between variables. Table 3 shows that any psychiatric diagnosis (OR = 1.14, *p* = 0.027, CI 95% [1.01,1.28]), ASD (OR = 1.28, *p* = 0.031, CI 95% [1.02,1.61]), conduct disorders (OR 1.49, *p* = <0.001, CI 95% [1.22, 1.81]), and PTSD (OR = 2.42, *p* = 0.017, CI 95% [1.17,4.99]) all remained significantly increased in children born with FIRS even after controlling for significant confounders **(**also see Table A3 for full models). ADHD diagnosis was borderline significant (OR = 1.19, *p* = 0.050, CI 95% [1.00, 1.42]). Any prescribed psychiatric medications also remained significantly different between the two groups (OR = 1.23, *p* = 0.046, CI 95% [1.00, 1.50]).

**Table 3:** Relationship between Fetal Inflammatory Response Syndrome (FIRS) and psychiatric diagnoses after controlling for potential confounders.

	Beta	Odds ratio	Odds ratio 95% CI	*t*-stat	*p*
Posttraumatic Stress Disorder (PTSD)	0.88	2.42	(1.17, 4.99)	2.40	0.017
Conduct disorders	0.40	1.49	(1.22, 1.81)	3.99	<0.001
Attention Deficit Hyperactivity Disorder (ADHD) medications (narcotic)	0.26	1.30	(0.98, 1.71)	1.85	0.064
Autism Spectrum Disorder (ASD)	0.25	1.28	(1.02, 1.61)	2.15	0.031
Any psychiatric medications	0.20	1.23	(1.00, 1.50)	2.00	0.046
Any ADHD medications	0.19	1.21	(0.96, 1.53)	1.65	0.100
ADHD	0.18	1.19	(1.00, 1.42)	1.96	0.050
Any psychiatric disorders	0.13	1.14	(1.01, 1.28)	2.21	0.027

**Fig. 1:**
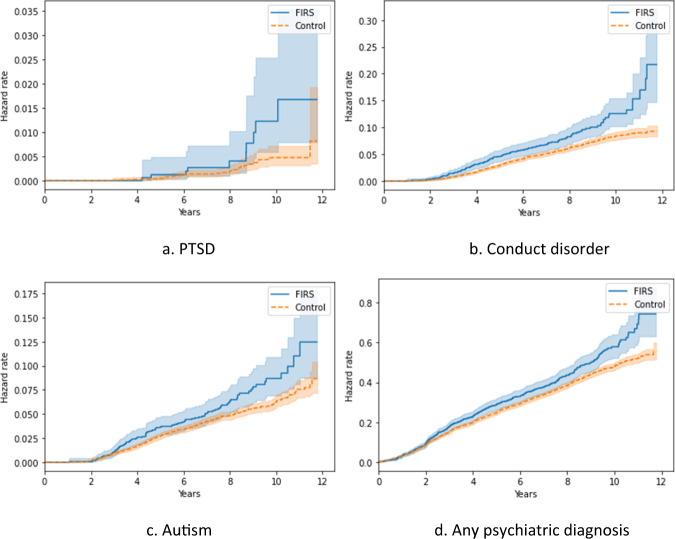
Time from birth to onset of PTSD, conduct disorder, autism spectrum disorder, and any psychiatric diagnosis, and in children both with and without Fetal Inflammatory Response Syndrome (FIRS). Nelson-Aalen cumulative hazard estimators for variables significantly different in FIRS and non-FIRS populations in Cox proportional hazards regression. Error bars are for *p* = 0.05.

Nelson-Aalen cumulative hazard estimators for psychiatric diagnoses are presented in Fig. 1. Using Cox proportional hazards models, we found a significantly increased risk of onsets of ASD (HR = 1.28, *p* = 0.028, CI 95% [1.03, 1.59]), conduct disorder (HR = 1.46, *p* < 0.001, CI 95% [1.21, 1.76]), PTSD (HR = 2.37, *p* = 0.019, CI 95% [1.15, 4.87]), and any psychiatric diagnosis (HR = 1.10, *p* = 0.038, CI 95% [1.01, 1.20]) in children born with FIRS compared to those without FIRS (Table 4, Table A4 for full models).

**Table 4:** Relationship between Fetal Inflammatory Response Syndrome (FIRS) and time from birth to onset of psychiatric diagnoses controlling for potential confounders.

	Hazard ratio	Hazard ratio 95% CI	*p*	Incidence Rate Ratio	Incidence Rate Ratio CI
Posttraumatic Stress Disorder (PTSD)	2.37	(1.15, 4.87)	0.019	2.50	(1.22, 5.13)
Conduct disorders	1.46	(1.21, 1.76)	<0.001	1.49	(1.24, 1.79)
Autism Spectrum Disorder (ASD)	1.28	(1.03, 1.59)	0.028	1.28	(1.03, 1.60)
ADHD medications (narcotic)	1.27	(0.97, 1.65)	0.082	1.34	(1.02, 1.74)
Any psychiatric medications	1.20	(1.00, 1.45)	0.056	1.25	(1.03, 1.51)
Any Attention Deficit Hyperactivity Disorder (ADHD) medications	1.18	(0.95, 1.47)	0.142	1.24	(1.00, 1.55)
ADHD	1.16	(0.99, 1.36)	0.072	1.21	(1.03, 1.42)
Any psychiatric disorders	1.10	(1.01, 1.20)	0.038	1.13	(1.03, 1.23)

### Sensitivity analyses

To examine the effects of data missingness on our results, we conducted sensitivity analyses and repeated all the above analyses: 1) using all records including those with less than 5 years of follow up; and 2) using the subset with at least 5 years of follow-up who had complete data on all variables. Using all available data, we compared 31,927 children without FIRS and 4851 children with FIRS with an average length of follow up of 4.3 years (IQR = 5.04), and 4.6 years (IQR = 5.35), respectively. In addition to the previously significant diagnoses (ADHD, ASD, conduct disorders, PTSD, and any psychiatric disorder), children born with FIRS were more likely to have anxiety disorders, bipolar disorders, and developmental delay (Table A5). Similar results were obtained regarding medications and in addition, children born with FIRS were significantly more likely to have been prescribed adrenergic medications compared to those without FIRS (Table A6). When controlling for potential confounders, the risk for ADHD, ASD, conduct disorders, PTSD, and bipolar disorders continued to be increased in FIRS using logistic regression models and the hazard for ASD, conduct disorders, PTSD, and bipolar disorders continued to be increased using Cox proportional hazards models (Tables A7 & A8).

We compared children with FIRS to those without FIRS on data missingness and found those with FIRS to have less complete data or more missingness compared to those without FIRS on various data sources (Table A9). For the subset with at least 5 years of follow-up who had complete data on all variables, similar results were obtained (ADHD, ASD, conduct disorders, PTSD, and any psychiatric disorder) with the additional result that children born with FIRS were more likely to have intellectual disability (Table A10). Similar results were also obtained for medications (Table A11). When controlling for significant confounders using logistic and (Table A12) and Cox proportional hazards models (Table A13), intellectual disability continued to be significant in both models.

## Discussion

We found an increased risk of neuropsychiatric disorders in children born with FIRS compared to those without FIRS. More specifically, there is increased incidence of ASD, ADHD, conduct disorder, PTSD, and any psychiatric disorder in these children, which was significant even after adjusting for maternal history of psychiatric disorders, maternal substance use during pregnancy, and maternal lifetime history of suicide attempts. While maternal alcohol, amphetamine, cannabis use disorder, and neonatal substance use disorder diagnoses were significantly increased in children born with FIRS compared to those without, children born with FIRS continued to show increased risk of psychiatric disorders even after adjusting for these potential confounders. These results suggest that inflammation of the placenta has a critical role in the pathogenesis of neuropsychiatric disorders during childhood, independent of many potential confounders.

We discuss these results in the context of its strengths and limitations. This study is the first to examine the impact of FIRS on risk for neuropsychiatric disorders using an EHR cohort. We included children born up to 2018 and had at least 5 years of follow-up and focused on childhood onset diagnoses and as such our results are not generalizable to adolescent onset diagnoses. It is also possible that some patients may be receiving care outside of the UPMC system and as such, we were not able to capture their offspring’s medical records. However, the region of this patient population is dominated by one child and adolescent psychiatric care system, reducing the possibility of patients being lost to follow up entirely, or follow up at other institutions. While the standard of practice at the women’s hospital used in the study is to only examine placentas which meet certain clinical criteria (e.g., low birth weight, intrauterine growth restriction, complications at delivery, poor newborn health), it is possible that abnormal placentas existed in the control group by virtue of not being examined microscopically. A recent study demonstrates that while many placentas have some level of inflammatory changes, they are almost always minor and unrelated to clinical outcomes [37]. In one study, only 6.7% of placentas assumed to be normal met criteria for FIRS upon closer microscopic examination [33]. Thus, the misclassification of FIRS placentas in the control group is likely to be small. We attempted to control for anti-inflammatory medications that may have inadvertently treated inflammation caused by FIRS. Additionally, some medications have both anti-infection and anti-inflammatory properties (e.g., rifampin), which could have treated FIRS (if caused by infectious etiology) and/or subsequent inflammation. We only considered maternal history of psychiatric disorders and were not able to account for paternal history.

Considering limitations related to EHR psychiatric outcomes, the DSM was revised from IV-TR to V during the study period. Liberalization and refining of some criteria, such as ASD, could have resulted in changes in the prevalence of diagnoses across the study period. However, it is likely that this refinement resulted in more accurate diagnosis, reflecting a possible underestimate of the true prevalence of these disorders [38]. Cultural changes occurred undoubtably and could have effects on parenting, social programs, and the societal milieu of children that were not captured in this study; however, these affected the groups of children with and without FIRS. We also had no information about patient’s history of trauma, given its lack of reliable ICD coding.

Considering the pathologic limitations to this study, the gold standard for FIRS diagnosis is cord blood (plasma Il-6 in fetal cord blood must be greater than 11 ng/ml); however, cord blood was not available for analysis in this study. Histologically, FIRS can be diagnosed by the presence of funisitis or chorionic vasculitis. While the criteria have been consistent throughout the study period (and since its inception as a diagnostic entity), this study did not account for the severity of fetal placental inflammation. Despite subspecialized gynecologic pathologists reviewing these records, there are variations among diagnoses that are rendered or emphasized for placental pathology reports, often with emphasis on clinically actionable data, such as chorioamnionitis. There is also the possibility that some diagnoses were placed in pathology report comment, and thus not captured using diagnostic codes. Funisitis and chorionic vasculitis are not completely specific for fetal inflammation, as both can be seen in other conditions such as chorioamnionitis.

We had no information about maternal infection during pregnancy and maternal stress in utero, which could result in increased maternal inflammation and immune responses. Direct and indirect evidence suggest that maternal immune activation is associated with increased risk of neuropsychiatric disorders. A Swedish registry study showed that fetal exposure to maternal infection increased the risk for autism and depression, but not bipolar or psychotic disorders [39]. Maternal stress in utero has known associations with psychiatric disease in offspring [40]. Maternal infections have also been directly associated with increased risk for ASD and schizophrenia in the offspring [41–43]. Maternal diseases that are associated with increased immune responses (e.g., autoimmune disorders, asthma) and maternal exposure to environmental and psychological stressors were previously found to be associated with increased risk for ASD and schizophrenia [40]. Maternal immune activation has been shown to affect such risk in the offspring through alterations in the prenatal environment, specifically through dysregulations in inflammatory pathways [44]. Animal models showed that IL-6 can directly cross the placenta and target the fetal brain and that a single injection of IL-6 in pregnant mice resulted in reduced social interaction and other behavioral abnormalities characteristic of ASD [45, 46]. Additionally, systemic maternal inflammation during pregnancy, especially elevated Il-6 levels, have been associated with cognitive impairment and development of psychiatric disease [47–50]. IL-1β was found to directly affect brain development through an alteration of the proliferation and differentiation of neural progenitor cells (NPCs) towards increasing gliogenesis and reducing neurogenesis [50]. Similarly, it has been shown that elevated maternal cytokines were associated with dendritic abnormalities characteristic of schizophrenia [9]. Indeed, increased IL-6, TNF-α, and other pro-inflammatory cytokines have been consistently implicated in psychiatric disorders, specifically, ASD, schizophrenia, and bipolar disorders [51–54]. Therapeutically, ongoing trials are using IL-6 inhibitors in pregnant women to treat FIRS in utero. Screening for FIRS and other risk factors during prenatal care is important to reduce the risk of childhood psychiatric disease.

Additional biological and molecular mechanisms are theorized to include the downstream effects of inflammation at a critical junction in neurodevelopment. As FIRS occurs later than 20 weeks of gestation, it occurs during a time of heightened vulnerability of prenatal brain development to environmental factors [20–23]. Alterations in epigenetic gene expression [55–57] could be an underlying mechanism by which FIRS (and its subsequent inflammation), alters the risk profile of a developing child. However, genetic factors could be also implicated in FIRS. Indeed, GWAS studies of ASD and ADHD show a shared genetic etiology along with schizophrenia and major depressive disorder [58] and the implicated genetic loci across these childhood- and adult-onset disorders were enriched in neuronal and immune pathways [59].

To our knowledge, this is the first study to examine fetal inflammatory syndrome as determined by pathological reports and its relationship with risk and onset of neuropsychiatric disorders. Children born with placenta meeting criteria for FIRS are at higher risk for neuropsychiatric disorders compared to children without FIRS. This study has potential implications for clinical care and prevention approaches. Children born with placenta meeting criteria for FIRS should be monitored closely for early identification and treatment. Future studies are needed to identify the biological mechanisms through which inflammation in general and FIRS in specific increases risk for neuropsychiatric disorders.

## Supplementary information

Appendix Table A1-13
