# Supplementary material for: Fetal inflammatory response and risk for psychiatric disorders
Source: Transl Psychiatry. 2023 Jun 24;13:224. doi: 10.1038/s41398-023-02505-3 (PMC10290670; doi:10.1038/s41398-023-02505-3)
Supplement: Supplementary file 1 — Appendix Table A1-13 [file 41398_2023_2505_MOESM1_ESM.docx]

**Table A1.** Groupings of diagnosis codes.

| **Diagnosis grouping** | **ICD9** | **ICD10** |
| --- | --- | --- |
| Attention Deficit Hyperactivity Disorder (ADHD) | 314.xx | F90.xx |
| Adjustment disorders | 309.4x, 309.28, 309.9x, 309.24, 309.3x, 309.89 | F43.20-25, F43.29 |
| Anxiety | 300.0x - 300.7x, excepting 300.3x | F41.xx |
| Autism Spectrum Disorder (ASD) | 299.xx | F84.xx |
| Conduct disorder | 312.xx | F91.xx |
| Depression | 296.2x - 296.3x, 311.xx | F32.xx - F33.xx |
| Obsessive Compulsive Disorders (OCD) | 300.3x | F42.xx |
| Posttraumatic Stress Disorder (PTSD) | 309.81 | F43.1x |
| Developmental delay | 315.xx | F81.9x, F89.xx |
| Intellectual disability | 317.xx - 319.xx, V62.89 | F7.xx, R41.83 |
| Any psychiatric disorder | 209.xx - 319.xx, V62.89, codes for any substance use disorder (below) | Fxx.xx, R41.83, Z72.0x |
| Suicide attempt | E950.xx - E959.xx | T14.91 |
| Any substance use disorder | 292.xx, 304.6x, 304.8x, 304.9x, 305.9x, 648.3x, V654.2x, codes for alcohol, amphetamine, cannabis, cocaine, hallucinogen, nicotine, opioid, and sedative use disorders (below) | F1x.xx |
| Alcohol use disorder | 291.xx, 303.xx, 305.0x, 535.3x, 357.5x, 425.5x, 571.0x - 571.3x, E860.0 | F10.xx |
| Amphetamine use disorder | 304.4x, 305.7x | F15.xx |
| Cannabis use disorder | 304.3x, 305.2x | F12.xx |
| Cocaine use disorder | 304.2x, 305.6x, 968.5x, 970.81, E938.5x | F14.xx |
| Hallucinogen use disorder | 304.5x, 305.3x, 969.6x, E854.1x, E939.6x | F16.xx |
| Nicotine use disorder | 305.1x | F17.xx, Z720.0x |
| Opioid use disorder | 304.0x, 304.7x, 305.5x, 965.0x, E850.0x, E935.0x | F11.xx |
| Sedative use disorder | 304.1x, 305.4x | F13.xx |
| Neonatal substance use | 779.5x, 760.70, 760.72, 779.89 | P96.1x, P04.49, F19.20 |

**Table A2.** Distributions of supplemental indicators of psychiatric diagnosis in FIRS and control subjects.

|  | **FIRS** | **Controls** | **test stat.** | **p** | **Odds Ratio** | **Odds Ratio**  **CI** |
| --- | --- | --- | --- | --- | --- | --- |
| ***Psychiatric medications*** | **N=1717** | **N=10915** |  | | | |
| Anxiety medications (non-benzodiazepine) | 0.2% (3) | 0.2% (17) | chi2 = 0.00 | 1.000 | 1.12 | (0.33, 3.83) |
| Anxiety medications (benzodiazepine) | 0.1% (1) | 0.1% (6) | chi2 = 0.00 | 1.000 | 1.06 | (0.13, 8.81) |
| Anticonvulsant medications (non-benzodiazepine) | 1.6% (27) | 1.4% (152) | chi2 = 0.23 | 0.634 | 1.13 | (0.75, 1.71) |
| Anticonvulsant medications (benzodiazepine) | 0.2% (3) | 0.2% (26) | chi2 = 0.06 | 0.811 | 0.73 | (0.22, 2.42) |
| Antipsychotic dopamine selective antagonist medications (DSA) | 0.2% (4) | 0.2% (24) | chi2 = 0.00 | 1.000 | 1.06 | (0.37, 3.06) |
| Antispsychotic medications (5HT) | 0.1% (2) | 0.1% (12) | chi2 = 0.00 | 1.000 | 1.06 | (0.24, 4.74) |
| Barbiturate medications | 0.1% (2) | 0.2% (17) | chi2 = 0.00 | 0.956 | 0.75 | (0.17, 3.24) |
| Selective Serotonin Reuptake Inhibitor (SSRI) | 0.9% (16) | 1.1% (119) | chi2 = 0.22 | 0.640 | 0.85 | (0.51, 1.44) |
| Serotonin antagonist and reuptake inhibitors (SARI) | 0.2% (4) | 0.1% (13) | chi2 = 0.71 | 0.400 | 1.96 | (0.64, 6.01) |
| Tricyclic Antidepressant (TCA) | 0.2% (3) | 0.2% (26) | chi2 = 0.06 | 0.811 | 0.73 | (0.22, 2.42) |
| ADHD (narcotic) | 4.5% (77) | 3.3% (362) | chi2 = 5.69 | **0.017** | 1.37 | (1.06, 1.76) |
| ADHD norepinephrine receptor inhibitor (NRI) | 0.4% (7) | 0.3% (30) | chi2 = 0.50 | 0.480 | 1.49 | (0.65, 3.39) |
| ADHD alpha 2 receptor agonist (A2RA) | 0.6% (11) | 0.6% (61) | chi2 = 0.06 | 0.806 | 1.15 | (0.60, 2.18) |
| Adrenergic medication | 3.8% (66) | 3.1% (335) | chi2 = 2.65 | 0.104 | 1.26 | (0.96, 1.65) |
| Any anxiety medications | 0.2% (4) | 0.2% (22) | chi2 = 0.00 | 1.000 | 1.16 | (0.40, 3.36) |
| Any anticonvulsant medications | 1.7% (29) | 1.5% (159) | chi2 = 0.40 | 0.528 | 1.16 | (0.78, 1.73) |
| Any antipsychotic medications | 0.3% (6) | 0.3% (32) | chi2 = 0.03 | 0.874 | 1.19 | (0.50, 2.86) |
| Any anti depressant medications (e.g. SSRI, SNRI, TCA) | 1.2% (21) | 1.4% (153) | chi2 = 0.23 | 0.632 | 0.87 | (0.55, 1.38) |
| Any ADHD medications | 6.6% (114) | 5.1% (561) | chi2 = 6.30 | **0.012** | 1.31 | (1.07, 1.62) |
| Any psychiatric medications | 8.8% (151) | 7.0% (763) | chi2 = 6.93 | **0.008** | 1.28 | (1.07, 1.54) |
| ***Psychiatric encounters (Yes/No)*** | **N=2041** | **N=12994** |  | | | |
| Inpatient psychiatric encounter | 0.2% (4) | 0.4% (48) | chi2 = 1.08 | 0.299 | 0.53 | (0.19, 1.47) |
| Outpatient psychiatric encounter | 1.4% (28) | 1.6% (207) | chi2 = 0.43 | 0.514 | 0.86 | (0.58, 1.28) |
| Any psychiatric encounter | 1.6% (32) | 1.9% (250) | chi2 = 1.03 | 0.310 | 0.81 | (0.56, 1.18) |
| ***Number of psychiatric encounters*** | **N=2041** | **N=12994** |  | | | |
| Number of psychiatric encounters | 0.049 +/- 0.517 | 0.051 +/- 0.503 | t = -0.14 | 0.891 | -- | -- |
| Number of inpatient psychiatric encounters | 0.006 +/- 0.167 | 0.014 +/- 0.315 | t = -1.03 | 0.304 | -- | -- |
| Number of outpatient psychiatric encounters | 0.043 +/- 0.49 | 0.037 +/- 0.37 | t = 0.61 | 0.539 | -- | -- |

**Table A3.** Full models for the relationship between Fetal Inflammatory Response Syndrome (FIRS) and psychiatric diagnosis controlling for significant confounders

|  | **Beta** | **Odds ratio** | **Odds ratio 95% CI** | **t-stat** | **p** |
| --- | --- | --- | --- | --- | --- |
| ***Attention Deficit Hyperactivity***  ***Disorder (ADHD)*** | | | | | |
| FIRS | 0.18 | 1.19 | (1.0, 1.42) | 1.96 | 0.050 |
| Anti-infection medication | 0.22 | 1.24 | (1.03, 1.49) | 2.29 | **0.022** |
| Anti-inflammatory medication | 0.37 | 1.44 | (1.27, 1.63) | 5.79 | **< 0.001** |
| Psychiatric diagnosis in mother | 0.36 | 1.43 | (1.25, 1.63) | 5.14 | **< 0.001** |
| Mother had suicide attempt | 0.26 | 1.30 | (0.81, 2.08) | 1.09 | 0.278 |
| Any substance exposure in utero | 0.21 | 1.24 | (1.01, 1.51) | 2.09 | **0.037** |
| Birth year | -0.31 | 0.73 | (0.71, 0.76) | -18.03 | **< 0.001** |
| ***Autism Spectrum Disorder (ASD)*** | | | | | |
| FIRS | 0.25 | 1.28 | (1.02, 1.61) | 2.15 | **0.031** |
| Anti-infection medication | 0.03 | 1.03 | (0.81, 1.3) | 0.23 | 0.818 |
| Anti-inflammatory medication | 0.30 | 1.35 | (1.15, 1.6) | 3.60 | **< 0.001** |
| Psychiatric diagnosis in mother | 0.26 | 1.30 | (1.09, 1.55) | 2.90 | **0.004** |
| Mother had suicide attempt | -0.55 | 0.58 | (0.23, 1.42) | -1.20 | 0.231 |
| Any substance exposure in utero | -0.17 | 0.84 | (0.62, 1.13) | -1.14 | 0.255 |
| Birth year | -0.14 | 0.87 | (0.83, 0.91) | -6.14 | **< 0.001** |
| ***Conduct disorder*** | | | | | |
| FIRS | 0.40 | 1.49 | (1.22, 1.81) | 3.99 | **< 0.001** |
| Anti-infection medication | 0.33 | 1.39 | (1.1, 1.75) | 2.81 | **0.005** |
| Anti-inflammatory medication | 0.23 | 1.26 | (1.09, 1.46) | 3.11 | **0.002** |
| Psychiatric diagnosis in mother | 0.28 | 1.32 | (1.13, 1.56) | 3.44 | **0.001** |
| Mother had suicide attempt | 0.13 | 1.14 | (0.64, 2.05) | 0.45 | 0.654 |
| Any substance exposure in utero | 0.13 | 1.14 | (0.9, 1.46) | 1.07 | 0.284 |
| Birth year | -0.18 | 0.83 | (0.8, 0.87) | -9.08 | **< 0.001** |
| ***Posttraumatic Stress Disorder (PTSD)*** | | | | | |
| FIRS | 0.88 | 2.42 | (1.17, 4.99) | 2.40 | **0.017** |
| Anti-infection medication | 0.71 | 2.04 | (0.62, 6.72) | 1.17 | 0.242 |
| Anti-inflammatory medication | 0.36 | 1.44 | (0.75, 2.76) | 1.09 | 0.276 |
| Psychiatric diagnosis in mother | 0.78 | 2.19 | (0.99, 4.84) | 1.93 | 0.053 |
| Mother had suicide attempt | 0.25 | 1.28 | (0.15, 10.86) | 0.23 | 0.818 |
| Any substance exposure in utero | 0.18 | 1.20 | (0.45, 3.17) | 0.36 | 0.716 |
| Birth year | -0.33 | 0.72 | (0.61, 0.86) | -3.66 | **< 0.001** |
| ***Any psychiatric diagnosis*** | | | | | |
| FIRS | 0.13 | 1.14 | (1.01, 1.28) | 2.21 | **0.027** |
| Anti-infection medication | 0.51 | 1.67 | (1.48, 1.87) | 8.56 | **< 0.001** |
| Anti-inflammatory medication | 0.39 | 1.47 | (1.36, 1.59) | 9.84 | **< 0.001** |
| Psychiatric diagnosis in mother | 0.14 | 1.15 | (1.06, 1.25) | 3.35 | **0.001** |
| Mother had suicide attempt | 0.28 | 1.32 | (0.95, 1.85) | 1.65 | 0.098 |
| Any substance exposure in utero | -0.03 | 0.97 | (0.85, 1.12) | -0.40 | 0.690 |
| Birth year | -0.07 | 0.93 | (0.91, 0.95) | -6.89 | **< 0.001** |
| ***ADHD medication (narcotic)*** | | | | | |
| FIRS | 0.26 | 1.30 | (0.98, 1.71) | 1.85 | 0.064 |
| Anti-infection medication | 0.12 | 1.13 | (0.84, 1.52) | 0.82 | 0.412 |
| Anti-inflammatory medication | 0.37 | 1.45 | (1.19, 1.78) | 3.62 | **< 0.001** |
| Psychiatric diagnosis in mother | 0.51 | 1.67 | (1.33, 2.11) | 4.34 | **< 0.001** |
| Mother had suicide attempt | 0.32 | 1.38 | (0.69, 2.78) | 0.91 | 0.365 |
| Any substance exposure in utero | 0.36 | 1.43 | (1.05, 1.93) | 2.30 | **0.021** |
| Birth year | -0.37 | 0.69 | (0.65, 0.73) | -13.31 | **< 0.001** |
| ***ADHD medication (any)*** | | | | | |
| FIRS | 0.19 | 1.21 | (0.96, 1.53) | 1.65 | 0.100 |
| Anti-infection medication | 0.04 | 1.04 | (0.82, 1.31) | 0.30 | 0.768 |
| Anti-inflammatory medication | 0.28 | 1.32 | (1.12, 1.56) | 3.30 | **0.001** |
| Psychiatric diagnosis in mother | 0.64 | 1.90 | (1.57, 2.31) | 6.52 | **< 0.001** |
| Mother had suicide attempt | 0.34 | 1.41 | (0.79, 2.49) | 1.16 | 0.245 |
| Any substance exposure in utero | 0.37 | 1.45 | (1.13, 1.85) | 2.94 | **0.003** |
| Birth year | -0.38 | 0.69 | (0.66, 0.72) | -16.33 | **< 0.001** |
| ***Any psychiatric medication*** | | | | | |
| FIRS | 0.20 | 1.23 | (1.0, 1.5) | 2.00 | **0.046** |
| Anti-infection medication | 0.01 | 1.01 | (0.82, 1.23) | 0.07 | 0.944 |
| Anti-inflammatory medication | 0.30 | 1.34 | (1.16, 1.55) | 4.05 | **< 0.001** |
| Psychiatric diagnosis in mother | 0.58 | 1.78 | (1.51, 2.09) | 6.89 | **< 0.001** |
| Mother had suicide attempt | 0.39 | 1.48 | (0.9, 2.45) | 1.54 | 0.124 |
| Any substance exposure in utero | 0.24 | 1.27 | (1.02, 1.59) | 2.11 | **0.035** |
| Birth year | -0.33 | 0.72 | (0.69, 0.75) | -16.62 | **< 0.001** |

**Table A4.** Full models for the relationship between Fetal Inflammatory Response Syndrome (FIRS) and time to from birth to onset of psychiatric diagnosis controlling for significant confounders

|  | **Hazard ratio** | **Hazard ratio 95% CI** | **p** |
| --- | --- | --- | --- |
| ***ADHD*** | | | |
| FIRS | 1.16 | (0.99, 1.36) | 0.072 |
| Anti-infection medication | 1.22 | (1.02, 1.45) | **0.026** |
| Anti-inflammatory medication | 1.43 | (1.27, 1.60) | **< 0.001** |
| Psychiatric diagnosis in mother | 1.39 | (1.22, 1.58) | **< 0.001** |
| Mother had suicide attempt | 1.27 | (0.83, 1.94) | 0.279 |
| Any substance exposure in utero | 1.24 | (1.03, 1.49) | **0.024** |
| Birth year | 1.00 | (0.80, 1.26) | 0.981 |
| ***Autism*** | | | |
| FIRS | 1.28 | (1.03, 1.59) | **0.028** |
| Anti-infection medication | 1.03 | (0.82, 1.30) | 0.789 |
| Anti-inflammatory medication | 1.35 | (1.15, 1.59) | **< 0.001** |
| Psychiatric diagnosis in mother | 1.28 | (1.08, 1.53) | **0.004** |
| Mother had suicide attempt | 0.58 | (0.24, 1.40) | 0.226 |
| Any substance exposure in utero | 0.85 | (0.63, 1.13) | 0.267 |
| Birth year | 1.17 | (0.81, 1.69) | 0.413 |
| ***Conduct disorder*** | | | |
| FIRS | 1.46 | (1.21, 1.76) | **< 0.001** |
| Anti-infection medication | 1.38 | (1.10, 1.72) | **0.005** |
| Anti-inflammatory medication | 1.25 | (1.09, 1.44) | **0.002** |
| Psychiatric diagnosis in mother | 1.31 | (1.13, 1.53) | **0.001** |
| Mother had suicide attempt | 1.13 | (0.65, 1.96) | 0.675 |
| Any substance exposure in utero | 1.13 | (0.90, 1.42) | 0.306 |
| Birth year | 0.93 | (0.68, 1.26) | 0.627 |
| ***PTSD*** | | | |
| FIRS | 2.37 | (1.15, 4.87) | **0.019** |
| Anti-infection medication | 2.02 | (0.62, 6.63) | 0.245 |
| Anti-inflammatory medication | 1.44 | (0.75, 2.76) | 0.269 |
| Psychiatric diagnosis in mother | 2.19 | (0.99, 4.85) | 0.054 |
| Mother had suicide attempt | 1.38 | (0.18, 10.29) | 0.756 |
| Any substance exposure in utero | 1.18 | (0.45, 3.12) | 0.732 |
| Birth year | 3.10 | (0.42, 22.94) | 0.268 |
| ***Any psychiatric diagnosis*** | | | |
| FIRS | 1.10 | (1.01, 1.20) | **0.038** |
| Anti-infection medication | 1.56 | (1.41, 1.72) | **< 0.001** |
| Anti-inflammatory medication | 1.40 | (1.31, 1.49) | **< 0.001** |
| Psychiatric diagnosis in mother | 1.10 | (1.03, 1.17) | **0.005** |
| Mother had suicide attempt | 1.27 | (0.99, 1.63) | 0.061 |
| Any substance exposure in utero | 0.99 | (0.89, 1.11) | 0.875 |
| Birth year | 1.13 | (0.97, 1.31) | 0.119 |
| ***ADHD medication (narcotic)*** | | | |
| FIRS | 1.27 | (0.97, 1.65) | 0.082 |
| Anti-infection medication | 1.13 | (0.84, 1.50) | 0.418 |
| Anti-inflammatory medication | 1.45 | (1.19, 1.76) | **< 0.001** |
| Psychiatric diagnosis in mother | 1.64 | (1.31, 2.06) | **< 0.001** |
| Mother had suicide attempt | 1.32 | (0.68, 2.57) | 0.418 |
| Any substance exposure in utero | 1.41 | (1.05, 1.89) | **0.021** |
| Birth year | 1.00 | (0.69, 1.45) | 0.993 |
| ***ADHD medication (any)*** | | | |
| FIRS | 1.18 | (0.95, 1.47) | 0.142 |
| Anti-infection medication | 1.03 | (0.83, 1.29) | 0.765 |
| Anti-inflammatory medication | 1.32 | (1.13, 1.55) | **0.001** |
| Psychiatric diagnosis in mother | 1.85 | (1.53, 2.23) | **< 0.001** |
| Mother had suicide attempt | 1.32 | (0.77, 2.25) | 0.311 |
| Any substance exposure in utero | 1.42 | (1.13, 1.80) | **0.003** |
| Birth year | 1.07 | (0.79, 1.46) | 0.654 |
| ***Any psychiatric medication*** | | | |
| FIRS | 1.20 | (1.00, 1.45) | 0.056 |
| Anti-infection medication | 1.02 | (0.84, 1.23) | 0.864 |
| Anti-inflammatory medication | 1.35 | (1.18, 1.54) | **< 0.001** |
| Psychiatric diagnosis in mother | 1.73 | (1.48, 2.02) | **< 0.001** |
| Mother had suicide attempt | 1.41 | (0.89, 2.23) | 0.142 |
| Any substance exposure in utero | 1.27 | (1.03, 1.56) | **0.027** |
| Birth year | 0.99 | (0.76, 1.28) | 0.920 |

**Table A5.** Distribution of psychiatric diagnoses in children both with and without FIRS using all available data.

|  | **FIRS (N=3405)** | **Control (N=28782)** | **χ2** | **p** | **Odds ratio** | **Odds ratio CI** |
| --- | --- | --- | --- | --- | --- | --- |
| ***Psychiatric diagnoses*** |  |  |  | | | |
| Attention Deficit Hyperactivity Disorder (ADHD) | 5.7% (194) | 4.1% (1180) | 18.63 | **< 0.001** | 1.41 | (1.21, 1.65) |
| Adjustment disorders | 3.0% (103) | 2.5% (731) | 2.65 | 0.103 | 1.20 | (0.97, 1.48) |
| Anxiety | 3.2% (110) | 2.5% (727) | 5.69 | **0.017** | 1.29 | (1.05, 1.58) |
| Autism Spectrum Disorder (ASD) | 3.7% (127) | 2.6% (761) | 13.0 | **< 0.001** | 1.43 | (1.18, 1.73) |
| Bipolar disorders | 0.2% (6) | 0.0% (14) | 6.06 | **0.014** | 3.63 | (1.39, 9.45) |
| Conduct disorder | 4.6% (156) | 2.8% (815) | 31.27 | **< 0.001** | 1.65 | (1.38, 1.96) |
| Depression | 0.4% (13) | 0.3% (79) | 0.88 | 0.347 | 1.39 | (0.77, 2.51) |
| Obsessive Compulsive Disorder (OCD) | 0.1% (4) | 0.2% (54) | 0.49 | 0.485 | 0.63 | (0.23, 1.73) |
| Psychosis | 0.1% (2) | 0.0% (5) | 0.87 | 0.351 | 3.38 | (0.66, 17.44) |
| Posttraumatic Stress Disorder (PTSD) | 0.3% (11) | 0.1% (34) | 7.75 | **0.005** | 2.74 | (1.39, 5.41) |
| Developmental delay | 7.9% (268) | 6.4% (1839) | 10.68 | **0.001** | 1.25 | (1.10, 1.43) |
| Intellectual disability | 0.4% (12) | 0.2% (56) | 2.89 | 0.089 | 1.81 | (0.97, 3.39) |
| Any substance use disorder | 0.6% (19) | 0.4% (105) | 2.48 | 0.115 | 1.53 | (0.94, 2.50) |
| Any psychiatric disorder | 24.8% (845) | 20.9% (6023) | 27.22 | **< 0.001** | 1.25 | (1.15, 1.35) |
| ***Suicide attempt*** |  |  |  | | | |
| Had suicide attempt | 0.0% (1) | 0.0% (1) | 0.44 | 0.507 | 8.46 | (0.53, 135.21) |

**Table A6.** Distributions of supplemental indicators of psychiatric diagnosis in FIRS and control subjects, using all available data.

|  | **FIRS** | **Control** | **test stat.** | **p** | **Odds ratio** | **Odds ratio CI** |
| --- | --- | --- | --- | --- | --- | --- |
| ***Psychiatric medication*** | **N=3169** | **N=21865** |  | | | |
| Anxiety (non-benzodiazepine) | 0.1% (3) | 0.1% (19) | chi2 = 0.00 | 1.000 | 1.09 | (0.32, 3.68) |
| Anxiety (benzodiazepine) | 0.0% (1) | 0.0% (10) | chi2 = 0.00 | 1.000 | 0.69 | (0.09, 5.39) |
| Anticonvulsant medication (non-benzodiazepine) | 1.1% (36) | 1.1% (238) | chi2 = 0.02 | 0.882 | 1.04 | (0.73, 1.49) |
| Anticonvulsant medication (benzodiazepine) | 0.3% (9) | 0.3% (61) | chi2 = 0.00 | 1.000 | 1.02 | (0.51, 2.05) |
| Antipsychotic (DSA) | 0.1% (4) | 0.1% (25) | chi2 = 0.00 | 1.000 | 1.10 | (0.38, 3.17) |
| Antispsychotic (5HT) | 0.1% (2) | 0.1% (12) | chi2 = 0.00 | 1.000 | 1.15 | (0.26, 5.14) |
| Barbiturate | 0.1% (4) | 0.1% (27) | chi2 = 0.00 | 1.000 | 1.02 | (0.36, 2.92) |
| Selective Serotonin Reuptake Inhibitor (SSRI) | 0.5% (16) | 0.6% (122) | chi2 = 0.06 | 0.804 | 0.90 | (0.54, 1.52) |
| Serotonin antagonist and reuptake inhibitors (SARI) | 0.1% (4) | 0.1% (13) | chi2 = 0.97 | 0.325 | 2.12 | (0.69, 6.52) |
| Tricycling Antidepressant (TCA) | 0.2% (6) | 0.2% (39) | chi2 = 0.00 | 1.000 | 1.06 | (0.45, 2.51) |
| Attention Deficit Hyperactivity Disorder (ADHD) (narcotic) | 2.4% (77) | 1.7% (367) | chi2 = 8.54 | **0.003** | 1.46 | (1.14, 1.87) |
| ADHD norepinephrine reuptake inhibitor (NRI) | 0.2% (7) | 0.1% (31) | chi2 = 0.68 | 0.409 | 1.56 | (0.69, 3.54) |
| ADHD alpha 2 receptor agonist (A2RA) | 0.3% (11) | 0.3% (61) | chi2 = 0.24 | 0.623 | 1.25 | (0.65, 2.37) |
| Adrenergic medication | 2.1% (66) | 1.5% (337) | chi2 = 4.79 | **0.029** | 1.36 | (1.04, 1.77) |
| Any anxiety medication | 0.1% (4) | 0.1% (28) | chi2 = 0.00 | 1.000 | 0.99 | (0.35, 2.81) |
| Any anticonvulsant medication | 1.3% (41) | 1.2% (260) | chi2 = 0.17 | 0.676 | 1.09 | (0.78, 1.52) |
| Any antipsychotic medication | 0.2% (6) | 0.2% (33) | chi2 = 0.07 | 0.786 | 1.25 | (0.53, 3.00) |
| Any depression medication | 0.8% (24) | 0.8% (169) | chi2 = 0.00 | 1.000 | 0.98 | (0.64, 1.50) |
| Any ADHD medication | 3.6% (114) | 2.6% (569) | chi2 = 9.95 | **0.002** | 1.40 | (1.14, 1.71) |
| Any psychiatric medication | 5.2% (166) | 4.1% (892) | chi2 = 8.90 | **0.003** | 1.30 | (1.10, 1.54) |
| ***Psychiatric encounters (dichotamous)*** | **N=4416** | **N=31384** |  | | | |
| Had inpatient psychiatric encounter | 0.1% (5) | 0.2% (48) | chi2 = 0.19 | 0.664 | 0.74 | (0.29, 1.86) |
| Had outpatient psychiatric encounter | 0.7% (29) | 0.7% (225) | chi2 = 0.12 | 0.726 | 0.92 | (0.62, 1.35) |
| Had any psychiatric encounter | 0.8% (34) | 0.9% (268) | chi2 = 0.23 | 0.629 | 0.90 | (0.63, 1.29) |
| ***Psychiatric encounters (count)*** | **N=4416** | **N=31384** |  | | | |
| Number of psychiatric encounters | 0.023 +/- 0.354 | 0.022 +/- 0.327 | t = 0.29 | 0.773 | -- | -- |
| Number of inpatient psychiatric encounters | 0.003 +/- 0.115 | 0.006 +/- 0.203 | t = -0.80 | 0.423 | -- | -- |
| Number of outpatient psychiatric encounters | 0.02 +/- 0.335 | 0.016 +/- 0.242 | t = 0.98 | 0.326 | -- | -- |

**Table A7.** Relationship between Fetal Inflammatory Response Syndrome (FIRS) and psychiatric diagnosis controlling for significant confounders using a dataset all available data

|  | **Beta** | **Odds ratio** | **Odds ratio 95% CI** | **t-stat** | **p** |
| --- | --- | --- | --- | --- | --- |
| Bipolar disorders | 1.12 | 3.08 | (1.18, 7.99) | 2.31 | **0.021** |
| Posttraumatic Stress Disorder (PTSD) | 0.90 | 2.46 | (1.23, 4.94) | 2.54 | **0.011** |
| Conduct disorder | 0.40 | 1.49 | (1.24, 1.79) | 4.20 | **< 0.001** |
| Attention Deficit Hyperactivity Disorder (ADHD) medication (narcotic) | 0.25 | 1.29 | (0.98, 1.70) | 1.82 | *0.069* |
| Autism Spectrum Disorder (ASD) | 0.23 | 1.26 | (1.03, 1.55) | 2.23 | **0.026** |
| ADHD | 0.20 | 1.22 | (1.02, 1.44) | 2.24 | **0.025** |
| Any ADHD medication | 0.19 | 1.21 | (0.96, 1.52) | 1.61 | 0.107 |
| Any psychiatric medication | 0.16 | 1.17 | (0.97, 1.41) | 1.65 | *0.098* |
| Adrenergic medication | 0.12 | 1.13 | (0.83, 1.52) | 0.77 | 0.441 |
| Developmental delay | 0.08 | 1.08 | (0.94, 1.25) | 1.11 | 0.269 |
| Anxiety | 0.07 | 1.07 | (0.86, 1.33) | 0.59 | 0.556 |
| Any psychiatric disorder | 0.06 | 1.07 | (0.97, 1.17) | 1.37 | 0.172 |

**Table A8.** Relationship between Fetal Inflammatory Response Syndrome (FIRS) and time from birth to onsets of psychiatric diagnosis controlling for significant confounders using all available data

|  | **Hazard ratio** | **Hazard ratio 95% CI** | **p** | **Incidence rate ratio** | **Incidence rate ratio CI** |
| --- | --- | --- | --- | --- | --- |
| Bipolar disorders | 2.99 | (1.15, 7.81) | **0.025** | 3.18 | (1.22, 8.29) |
| Posttraumatic Stress Disorder (PTSD) | 2.45 | (1.23, 4.88) | **0.011** | 2.64 | (1.33, 5.25) |
| Conduct disorders | 1.45 | (1.22, 1.73) | **< 0.001** | 1.51 | (1.26, 1.80) |
| Attention Deficit Hyperactivity Disorder (ADHD) medication (narcotic) | 1.24 | (0.95, 1.62) | 0.107 | 1.35 | (1.04, 1.76) |
| Autism Spectrum Disorder (ASD) | 1.23 | (1.01, 1.51) | **0.037** | 1.27 | (1.04, 1.55) |
| ADHD | 1.17 | (1.00, 1.37) | *0.054* | 1.25 | (1.07, 1.46) |
| Any ADHD medication | 1.16 | (0.93, 1.44) | 0.193 | 1.26 | (1.01, 1.56) |
| Any psychiatric medication | 1.15 | (0.96, 1.37) | 0.133 | 1.21 | (1.01, 1.45) |
| Developmental delay | 1.11 | (0.97, 1.27) | 0.121 | 1.11 | (0.97, 1.27) |
| Adrenergic medication | 1.08 | (0.81, 1.45) | 0.586 | 1.19 | (0.89, 1.59) |
| Anxiety | 1.04 | (0.85, 1.29) | 0.688 | 1.11 | (0.90, 1.37) |
| Any psychiatric disorder | 1.04 | (0.96, 1.12) | 0.320 | 1.06 | (0.99, 1.15) |

**Table A9.** Difference in diagnosis rates between subjects with and without complete data, for subjects with at least 5 years of data.

|  | **Has missing data (N=2023)** | **No missing data (N=12110)** | **χ2** | **p** | **Odds ratio** | **Odds ratio CI** |
| --- | --- | --- | --- | --- | --- | --- |
| Fetal Inflammatory Response Syndrome (FIRS) | 20.2% (598) | 12.0% (1457) | 132.91 | **< 0.001** | 0.54 | (0.49, 0.60) |
| Attention Deficit Hyperactivity Disorder (ADHD) | 5.6% (114) | 10.0% (1211) | 38.36 | **< 0.001** | 1.86 | (1.53, 2.27) |
| Adjustment disorders | 3.0% (60) | 5.6% (680) | 23.99 | **< 0.001** | 1.95 | (1.49, 2.55) |
| Anxiety | 2.8% (56) | 6.1% (738) | 35.54 | **< 0.001** | 2.28 | (1.73, 3.00) |
| Autism Spectrum Disorder (ASD) | 3.3% (67) | 5.2% (628) | 12.62 | **< 0.001** | 1.60 | (1.24, 2.06) |
| Conduct Disorders | 3.4% (69) | 6.6% (801) | 30.24 | **< 0.001** | 2.01 | (1.56, 2.58) |
| Depression | 0.3% (7) | 0.5% (64) | 0.82 | 0.366 | 1.53 | (0.70, 3.34) |
| Obsessive Compulsive Disorder (OCD) | 0.3% (7) | 0.4% (43) | 0.00 | 1.000 | 1.03 | (0.46, 2.28) |
| Posttraumatic Stress Disorder (PTSD) | 0.1% (2) | 0.3% (39) | 2.26 | 0.132 | 3.26 | (0.79, 13.53) |
| Developmental delay | 5.9% (119) | 15.2% (1846) | 126.12 | **< 0.001** | 2.88 | (2.38, 3.49) |
| Intellectual disability | 0.3% (6) | 0.4% (48) | 0.23 | 0.632 | 1.34 | (0.57, 3.13) |
| Any psychiatric disorders | 20.3% (410) | 34.4% (4163) | 157.02 | **< 0.001** | 2.06 | (1.84, 2.31) |
| Suicide attempt | 0.0% (0) | 0.0% (2) | 0.00 | 1.000 | - | - |

**Table A10.** Distribution of psychiatric diagnoses in children both with and without FIRS, using the subset of subjects with at least 5 years of data and no missing data.

|  | **FIRS (N=1457)** | **Control (N=10653)** | **χ2** | **p** | **Odds ratio** | **Odds ratio CI** |
| --- | --- | --- | --- | --- | --- | --- |
| ***Psychiatric diagnoses*** |  |  |  | | | |
| Attention Deficit Hyperactivity Disorder (ADHD) | 11.8% (172) | 9.8% (1039) | 5.77 | **0.016** | 1.24 | (1.04, 1.47) |
| Adjustment disorders | 5.7% (83) | 5.6% (597) | 0.01 | 0.934 | 1.02 | (0.80, 1.29) |
| Anxiety | 6.9% (100) | 6.0% (638) | 1.56 | 0.211 | 1.16 | (0.93, 1.44) |
| Autism Spectrum Disorder (ASD) | 6.5% (94) | 5.0% (534) | 5.1 | **0.024** | 1.31 | (1.04, 1.64) |
| Bipolar disorders | 0.3% (5) | 0.1% (13) | 2.86 | 0.091 | 2.82 | (1.00, 7.92) |
| Conduct disorder | 9.3% (135) | 6.3% (666) | 18.36 | **< 0.001** | 1.53 | (1.26, 1.86) |
| Depression | 0.5% (7) | 0.5% (57) | 0.01 | 0.939 | 0.90 | (0.41, 1.97) |
| Obsessive Compulsive Disorder (OCD) | 0.2% (3) | 0.4% (40) | 0.62 | 0.432 | 0.55 | (0.17, 1.77) |
| Psychosis | 0.1% (2) | 0.0% (4) | 0.95 | 0.329 | 3.66 | (0.67, 20.00) |
| Posttraumatic Stress Disorder (PTSD) | 0.7% (10) | 0.3% (29) | 5.62 | **0.018** | 2.53 | (1.23, 5.21) |
| Developmental delay | 16.5% (241) | 15.1% (1605) | 2.04 | 0.153 | 1.12 | (0.96, 1.30) |
| Intellectual disability | 0.8% (11) | 0.3% (37) | 4.41 | **0.036** | 2.18 | (1.11, 4.29) |
| Any substance use disorder | 0.5% (7) | 0.6% (59) | 0.03 | 0.867 | 0.87 | (0.40, 1.90) |
| Any psychiatric disorder | 36.1% (593) | 31.9% (3980) | 11.92 | **0.001** | 1.21 | (1.09, 1.35) |
| ***Suicide attempt*** |  |  |  | | | |
| Had suicide attempt | 0.1% (1) | 0.0% (1) | 0.32 | 0.573 | 7.32 | (0.46, 117.03) |

**Table A11.** Distributions of supplemental indicators of psychiatric diagnoses in FIRS and control subjects, using the subset of subjects with at least 5 years of data and no missing data.

|  | **FIRS** | **Control** | **test stat.** | **p** | **Odds ratio** | **Odds ratio CI** |
| --- | --- | --- | --- | --- | --- | --- |
| ***Psychiatric medication*** | **N=1457** | **N=10653** |  | | | |
| Anxiety (non-benzodiazepine) | 0.2% (3) | 0.2% (16) | chi2 = 0.02 | 0.880 | 1.37 | (0.40, 4.71) |
| Anxiety (benzodiazepine) | 0.1% (1) | 0.1% (6) | chi2 = 0.00 | 1.000 | 1.22 | (0.15, 10.13) |
| Anticonvulsant medication (non-benzodiazepine) | 1.7% (25) | 1.4% (146) | chi2 = 0.86 | 0.353 | 1.26 | (0.82, 1.93) |
| Anticonvulsant medication (benzodiazepine) | 0.1% (2) | 0.2% (25) | chi2 = 0.20 | 0.658 | 0.58 | (0.14, 2.47) |
| Antipsychotic dopamine selective antagonist (DSA) | 0.2% (3) | 0.2% (24) | chi2 = 0.00 | 1.000 | 0.91 | (0.27, 3.04) |
| Antispsychotic (5HT) | 0.1% (1) | 0.1% (12) | chi2 = 0.00 | 0.956 | 0.61 | (0.08, 4.69) |
| Barbiturate | 0.1% (2) | 0.2% (17) | chi2 = 0.00 | 1.000 | 0.86 | (0.20, 3.73) |
| Selective Serotonin Reuptake Inhibitor (SSRI) | 0.9% (13) | 1.1% (118) | chi2 = 0.37 | 0.541 | 0.80 | (0.45, 1.43) |
| Serotonin antagonist and reuptake inhibitors (SARI) | 0.2% (3) | 0.1% (12) | chi2 = 0.30 | 0.581 | 1.83 | (0.52, 6.49) |
| Tricyclic Antidepressant (TCA) | 0.1% (2) | 0.2% (26) | chi2 = 0.26 | 0.613 | 0.56 | (0.13, 2.37) |
| ADHD (narcotic) | 4.5% (65) | 3.3% (354) | chi2 = 4.64 | **0.031** | 1.36 | (1.04, 1.78) |
| ADHD norepinephrine reuptake inhibitor (NRI) | 0.5% (7) | 0.3% (29) | chi2 = 1.24 | 0.266 | 1.77 | (0.77, 4.04) |
| ADHD alpha 2 receptor agonist (A2RA) | 0.8% (11) | 0.6% (60) | chi2 = 0.51 | 0.474 | 1.34 | (0.70, 2.56) |
| Adrenergic medication | 3.6% (53) | 3.1% (329) | chi2 = 1.09 | 0.296 | 1.18 | (0.88, 1.59) |
| Any anxiety medication | 0.3% (4) | 0.2% (21) | chi2 = 0.09 | 0.762 | 1.39 | (0.48, 4.07) |
| Any anticonvulsant medication | 1.9% (27) | 1.4% (153) | chi2 = 1.25 | 0.264 | 1.30 | (0.86, 1.96) |
| Any antipsychotic medication | 0.3% (4) | 0.3% (32) | chi2 = 0.00 | 1.000 | 0.91 | (0.32, 2.59) |
| Any depression medication | 1.2% (17) | 1.4% (151) | chi2 = 0.42 | 0.517 | 0.82 | (0.50, 1.36) |
| Any ADHD medication | 6.5% (94) | 5.2% (549) | chi2 = 4.04 | **0.044** | 1.27 | (1.01, 1.59) |
| Any psychiatric medication | 8.8% (128) | 7.0% (748) | chi2 = 5.68 | **0.017** | 1.28 | (1.05, 1.55) |
| ***Psychiatric encounters (dichotamous)*** | **N=1457** | **N=10653** |  | | | |
| Had inpatient psychiatric encounter | 0.3% (4) | 0.3% (37) | chi2 = 0.04 | 0.835 | 0.79 | (0.28, 2.22) |
| Had outpatient psychiatric encounter | 1.5% (22) | 1.7% (176) | chi2 = 0.08 | 0.771 | 0.91 | (0.58, 1.43) |
| Had any psychiatric encounter | 1.8% (26) | 2.0% (209) | chi2 = 0.13 | 0.719 | 0.91 | (0.60, 1.37) |
| ***Psychiatric encounters (count)*** | **N=1457** | **N=10653** |  | | | |
| Number of psychiatric encounters | 0.056 +/- 0.576 | 0.053 +/- 0.528 | t = 0.22 | 0.823 | -- | -- |
| Number of inpatient psychiatric encounters | 0.009 +/- 0.198 | 0.013 +/- 0.322 | t = -0.47 | 0.641 | -- | -- |
| Number of outpatient psychiatric encounters | 0.047 +/- 0.542 | 0.04 +/- 0.395 | t = 0.63 | 0.526 | -- | -- |

**Table A12.** Relationship between Fetal Inflammatory Response Syndrome (FIRS) and psychiatric diagnoses controlling for potential confounders, using the subset of subjects with at least 5 years of data and no missing data.

|  | **Beta** | **Odds ratio** | **Odds ratio 95% CI** | **t-stat** | **p** |
| --- | --- | --- | --- | --- | --- |
| Posttraumatic stress disorder (PTSD) | 0.88 | 2.42 | (1.18, 4.99) | 2.40 | **0.016** |
| Intellectual disability | 0.69 | 1.99 | (1.00, 3.94) | 1.97 | **0.049** |
| Conduct disorder | 0.40 | 1.49 | (1.23, 1.81) | 4.00 | **< 0.001** |
| Attention Deficent Hyperactivity Disorder (ADHD) medication (narcotic) | 0.26 | 1.30 | (0.99, 1.71) | 1.85 | 0.064 |
| Autism Spectrum Disorder (ASD) | 0.25 | 1.29 | (1.02, 1.61) | 2.16 | **0.031** |
| Any psychiatric medication | 0.20 | 1.23 | (1.00, 1.50) | 2.00 | **0.046** |
| Any ADHD medication | 0.19 | 1.21 | (0.96, 1.53) | 1.65 | 0.100 |
| ADHD | 0.18 | 1.19 | (1.00, 1.42) | 1.97 | **0.049** |
| Any psychiatric disorder | 0.13 | 1.14 | (1.02, 1.28) | 2.26 | **0.024** |

**Table A13.** Relationship between Fetal Inflammatory Response Syndrome (FIRS) and time from birth to onsets of psychiatric diagnosis in Cox proportional hazards model, using the subset of subjects with at least 5 years of data and no missing data.

|  | **Hazard ratio** | **Hazard ratio 95% CI** | **p** | **Incidence rate ratio** | **Incidence rate ratio CI** |
| --- | --- | --- | --- | --- | --- |
| Posttraumatic Stress Disorder (PTSD) | 2.37 | (1.15, 4.88) | **0.019** | 2.50 | (1.22, 5.13) |
| Intellectual disability | 1.99 | (1.01, 3.92) | **0.047** | 2.16 | (1.10, 4.23) |
| Conduct disorder | 1.46 | (1.22, 1.76) | **< 0.001** | 1.49 | (1.24, 1.80) |
| Autism Spectrum Disorder (ASD) | 1.28 | (1.03, 1.59) | **0.028** | 1.29 | (1.03, 1.60) |
| Attention Deficet Hyperactivity Disorder (ADHD) medication (narcotic) | 1.27 | (0.97, 1.65) | 0.081 | 1.34 | (1.03, 1.74) |
| Any psychiatric medication | 1.20 | (1.00, 1.45) | 0.056 | 1.25 | (1.04, 1.51) |
| Any ADHD medication | 1.18 | (0.95, 1.47) | 0.141 | 1.25 | (1.00, 1.55) |
| ADHD | 1.16 | (0.99, 1.36) | 0.071 | 1.21 | (1.03, 1.42) |
| Any psychiatric disorder | 1.10 | (1.01, 1.21) | **0.033** | 1.13 | (1.03, 1.24) |
